# Analysis of the Influence of Entrepreneur’s Psychological Capital on Employee’s Innovation Behavior Under Leader-Member Exchange Relationship

**DOI:** 10.3389/fpsyg.2020.01853

**Published:** 2020-07-31

**Authors:** Tingyi Li, Wei Liang, Zhijian Yu, Xin Dang

**Affiliations:** ^1^School of Business Administration, Wonkwang University, Iksan, South Korea; ^2^College of Humanities, Shandong Agriculture and Engineering University, Jinan, China; ^3^School of Agricultural Economics and Rural Development, Renmin University of China, Beijing, China; ^4^College of International Business, Shandong Technology and Business University, Yantai, China

**Keywords:** LMX relationship, psychological capital of leaders, employee innovation behavior, hierarchical regression method, multiple regression analysis

## Abstract

How to make use of leaders’ psychological capital to improve the innovation behavior of employees has become an important issue for the talent management of enterprises today, and it is also the goal that enterprises must pursue if they want to stand out in fierce competition. Therefore, a total of 154 enterprises in a high-tech area were selected for questionnaire survey in this study. The correlation between leader-member exchange (LMX) relationship (emotion, loyalty, contribution, and professional respect), leaders’ psychological capital (confidence, hope, optimism, and tenacity), and employees’ innovation behaviors were analyzed based on multivariate regression. Hierarchical regression method was used to examine the mediating effect of the LMX. It was found that confidence, toughness, and contribution were significantly positively correlated with employee innovation behavior (*p* < 0.001). The positive correlation between hope, optimism, emotion, and loyalty with employees’ innovation behavior was significant (*p* < 0.05). Besides, emotion, loyalty, and contribution had mediating effects on the leaders’ psychological capital and the innovation behavior of employees. In conclusion, leaders’ psychological capital can have a significant positive effect on the innovation behavior of employees directly, and it can also have an indirect positive effect on the innovation behavior of employees by maintaining high quality LMX.

## Introduction

With the development of economic globalization, the biggest competitive advantage of a modern enterprise is not cost saving and product quality, nor talent, but talent management and organization (Wang et al., 2014; Trinchero et al., 2019). As Ren Zhengfei, President of Huawei, once said, “Talents are not the core competitiveness of Huawei, but the ability to effectively manage talents is the core competitiveness of an enterprise. This is the core talent concept that enables the Huawei team to maintain a high level of creativity, and it is also what we need to learn from now” (Arasli et al., 2020). When it comes to the management of employees, leaders must be involved. A good entrepreneur’s psychological capital can not only improve the leader’s self-consciousness, self-adjustment, and self-development ability but also have a trickle-down effect on the subordinate’s psychological state, and enhance the subordinate’s psychological capital (Niu et al., 2018; Shen et al., 2019; Wu and Wu, 2019). Psychological capital is a concept proposed and extended to the field of human resources by Luthans, a famous American scholar, in 2004. As a special intangible resource, it has become one of the highlights among many factors affecting the occurrence of enterprises. It is a core psychological element beyond human capital and social capital and can promote personal growth and performance improvement (Chen, 2020; Montani et al., 2020). Therefore, in actual business operation, it is very important to pay attention to the development of psychological capital of leaders and employees.

In recent years, according to many studies, psychological capital reveals that in order to improve the overall performance and competitive advantage of enterprises, leaders can improve the innovation performance of employees by improving their psychological capital (Lee, 2019; Muldoon et al., 2019; Yoon and Kim, 2019). Mishra et al. (2019) investigated the relationship of psychological capital and leadership support in promoting innovative work behavior among 398 service sector employees, and found that the leader support is directly related to the innovation behavior, and the psychological capital completely mediated between the leader support and the innovation work behavior. Slåtten et al. (2019) examined the relationship between psychological capital, social capital, and job performance and found that psychological capital was directly related to innovation behavior, job involvement, and sales performance, and both structural and relational dimensions were directly related to psychological dimensions. Safavi and Bouzari (2019) collected data related to psychological capital, occupational innovation, and occupational competency characteristics from 193 front-line employees and concluded that there was a significant relationship between psychological capital and occupational innovation, and occupational adaptability regulated the relationship between psychological capital and occupational innovation. Leader-member exchange (LMX) (Qureshi et al., 2020), an important leadership theory, has attracted much attention in recent years. As one of the major factors in the organization, theoretical or empirical studies on the relationship between superior and subordinate in Chinese enterprises need to be further developed. At present, there are few reports on the exchange of leader-member relationship in China. Therefore, it is novel to start with LMX theory.

To sum up, there are many researches on the innovation ability of employees in enterprises, but most of them focus on the psychological capital of employees, and there is little analysis from the perspective of psychological capital of leaders. Based on this, 154 enterprises in high-tech area were selected for questionnaire survey. The differences of different demographic variables LMX, leader’s psychological capital, and employee’s innovation behavior were analyzed. The correlation between LMX, leader’s psychological capital, and employee’s innovation behavior was analyzed by multiple regression. The hierarchical regression method was used to examine the mediating effect of the LMX between the psychological capital of the leader and the innovation behavior of the employee, so as to comprehensively evaluate the influence of the psychological capital of the leader on the innovation behavior of the employee.

## Literature Review

At present, many domestic and foreign scholars have analyzed the relationship between LMX and the innovation of employees. Carnevale et al. (2017) made a retrospective analysis of the research results of LMX and sound (37 samples), creativity (53 samples), and innovative behavior (29 samples) and concluded that LMX was more associated with innovative behavior than it was with sound or creativity. Lukes and Stephan (2017) developed a model of employee innovation behavior and conceptualized it as a multifaceted behavior that was different from the output of innovation, and found that the behavior of innovation output changed greatly after the introduction of high-quality LMX relationship. Cha and Borchgrevink (2018) collected data of 452 employees working in 31 different service organizations, and the multi-group structural equation model analysis showed that the LMX relationship had a great influence on employee innovation. Bruccoleri and Riccobono (2018) discussed the complex influence of objective practice management on employees’ innovative behaviors and found that the unconscious incentive path linked the quality of leader-follower relationship with employees’ innovative behaviors through appropriate performance evaluation strategies. However, studies on the relationship between psychological capital and employee innovation were almost limited to employees’ own psychological capital (Bruccoleri and Riccobono, 2018). Kim et al. (2018) proposed to take psychological capital as part of the mediating variable of the impact of psychological contract violation on service innovation behavior, and the structural equation model revealed that the development of psychological capital activated employees’ service innovation behavior. Guo et al. (2018) discussed the influence of employees’ psychological capital on their creativity, and drew a conclusion that when psychological capital was relatively high, it can regulate employees’ fear and creativity. Mishra et al. (2019) examined the relationship between two-way work-family enrichment, psychological capital, and executive support in promoting innovative work behavior, and found that psychological capital was completely mediated between bidirectional enrichment and innovative work behavior.

To sum up, most of the current researches on psychological capital were focused on the employees themselves, while the leader’s psychological capital was rarely involved. There was little research on LMX relationship, leader’s psychological capital, and employee’s innovative behavior. Therefore, based on the LMX relationship, the regulating mechanism of the psychological capital of leaders on the innovation behavior of employees was explored, so as to develop the theoretical basis for improving the innovation of employees.

## Materials and Methods

### Samples and Data Collection

At the individual level, the relationship among leaders’ psychological capital, employees’ innovation behavior, and LMX relationship was explored. Considering that knowledge-based young people are more likely to accept new things, good at learning, and have many new ideas, leaders prefer young knowledge-based employees with certain work experience in the sample selection. In this study, 154 enterprises in a high-tech region were randomly selected through the Internet to issue questionnaires. A total of 416 questionnaires were distributed and 378 were recovered. After invalid data was removed, there were 345 valid copies, with a recovery efficiency of 82.93%.

This survey involved a wide range of industries, large and small enterprises, and had a certain universality and promotion significance. As shown in Table 1, in terms of the nature of enterprises, private enterprises account for the most (42.18%), followed by state-owned enterprises (36.88%). In terms of the industries to which the enterprises belong, the Internet industry accounted for the most (42.07%), followed by the service industry (26.37%). In terms of the number of employees, the enterprises with 1–300 employees account for the most (51.68%), followed by the enterprises with 300–999 employees (30.72%); in other words, there were mainly small and medium-sized enterprises.

**TABLE 1:** Basic information of tested enterprises.

Variables	Classification	Number of samples	Proportion (%)
Nature of the enterprises	Private	65	42.18
	Foreign-invested	32	20.94
	State-owned	57	36.88
Industries the enterprises belong to	Internet	65	42.07
	Real state	35	22.84
	Service	41	26.37
	Other	13	8.72
Number of employees	1–300	80	51.68
	300–999	47	30.72
	More than 999	27	17.60

The demographic characteristics of the selected samples were shown in Table 2. Among them, there were 106 managers and 249 ordinary employees. In terms of gender, the ratios of males (52.96%) and females (47.04%) were not much different. In terms of age, employees were generally under 40 years old. The proportion of employees aged 18–30 years was the largest (46.38%), followed by those aged 30–40 years (37.41%). In terms of education background, employees with bachelor’s degree accounted for the most (53.84%), and those with master’s degree or above accounted for the least (18.30%). In terms of marriage, the number of married employees was higher, accounting for 71.36%, while the number of unmarried employees was lower (28.64%).

**TABLE 2:** Basic information of the tested employees.

Variable	Classification	Number of the samples	Proportion (%)
Staff	Manager	106	29.86
	Ordinary employee	249	70.14
Age	18–30	160	46.38
	30–40	129	37.41
	40–55	56	16.21
Education background	Junior college	96	27.86
	Bachelor degree	186	53.84
	Master degree and above	63	18.30
Gender	Male	162	47.04
	Female	183	52.96
Marriage status	Married	246	71.36
	Unmarried	99	28.64
			

### LMX Theory

**FIGURE 1:**
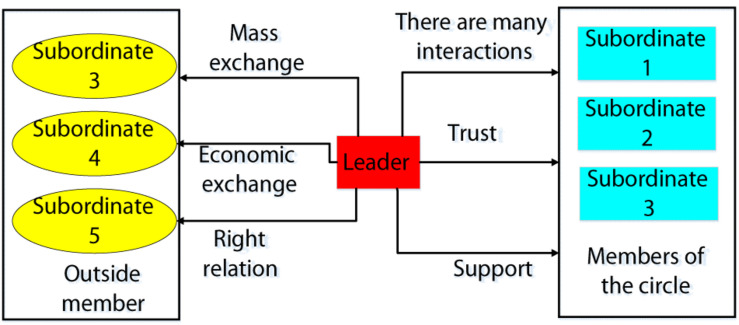
Structure diagram of LMX theory.

Leader-member exchange theory (Choi, 2020) was first proposed by George Graen and Uhl-bien in 1976. It refers to a kind of close relationship between leaders and employees cultivated through a series of exploration, observation, and interaction. As shown in Figure 1, when the relationship between the leader and the employee is relatively close, that is, the high-quality LMX relationship and the connection between the two parties not only depends on the basic economic exchange, but also involves the emotional exchange. Employees have more autonomy and development opportunities, they are more active and active when they obey leaders, and they can use their best talents to complete work tasks. They are called “in the circle” members. The low-quality LMX relationship is mainly based on the exchange of materials based on the power system. The psychological distance between superiors and subordinates is relatively long, and they seldom get extra reward and opportunities from leaders. Such employees are called “out-of-circle” members. Psychological research has found that the level of LMX relationship affects the work efficiency and innovation behavior of employees to a certain extent.

### Establishment of the Relation Model

Although there are many researches on the innovation behavior of employees, few analyses on the relationship between leaders and subordinates in China have been made. What role does the relationship exchange between leaders and employees play in employee innovation behavior? Does the psychological capital of leaders directly influence the innovation behavior of employees significantly? These relationships are unclear.

**FIGURE 2:**
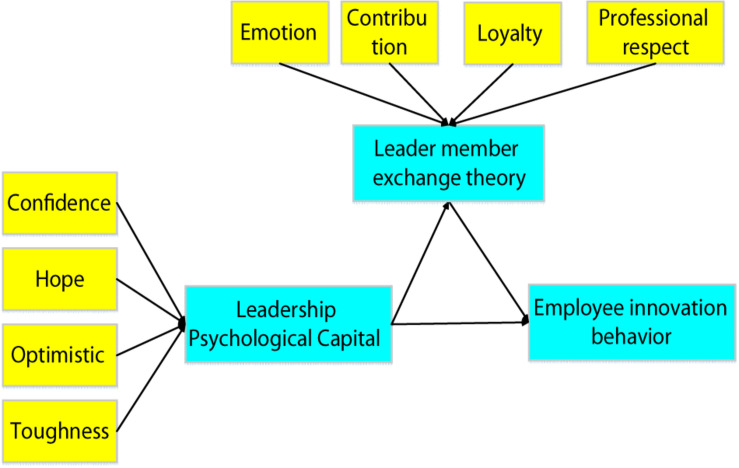
The relational model of leader psychological capital, employee innovation behavior, and LMX.

Some psychology-based studies have shown that leaders with a high level of psychological capital are more likely to gain the respect and support of their subordinates, and thus are more likely to establish a good relationship between superiors and subordinates. As a project that requires a lot of time and energy, innovation often requires individuals to have higher self-confidence and ability to work under pressure. A good leader can give enough trust and encouragement to subordinates and provide a good innovation environment and conditions for employees (Gu et al., 2019). Based on this, a relationship model among the three variables of leader psychological capital, employee innovation behavior, and LMX relationship was established. In this model, the psychological capital of leaders (confidence, hope, optimism, and toughness) can directly influence the innovation behavior of employees, and it can also indirectly influence the innovation behavior of employees by changing the exchange relationship of leaders (emotion, loyalty, contribution, and professional respect), as shown in Figure 2 below.

### Scale Tools

#### Leaders’ Psychological Capital Scale

The leaders’ psychological capital scale proposed by Luthans et al. (2010) and Liao et al. (2019) was adopted to evaluate leaders. The scale contained four dimensions of confidence, hope, optimism, and resilience, with a total of 20 items. Likert 5-grade rating system was adopted, in which 1–5 points, respectively indicated *strongly disagree*, *disagree*, *neutral*, *agree*, and *strongly agree*. The higher the score was, the higher the psychological capital level of the leader was. The internal consistency reliability coefficients of the scale with the four dimensions of the scale, namely, confidence, hope, optimism, and toughness were 0.871, 0.858, 0.931, and 0.915, respectively. According to the reliability and validity test, the Cronbach’s coefficient α was 0.872, and each test question was significantly correlated with the total score at the level of 0.01 (bilateral).

#### Employee Innovation Behavior Scale

Employee Innovation Behavior scale was a concept containing many contents. From the perspective of individual traits, it is a willingness to change the *status quo* of individuals. The employee innovation behavior scale (Lee, 2019) proposed by Zhang Zhengang in 2016 was adopted to evaluate the innovation of enterprise employees. This scale was a single dimension scale, which conformed to the actual situation of Chinese enterprises. There were eight test items in total. Likert 5-grade rating system was adopted, and 1–5 points, respectively indicated *strongly disagree*, *disagree*, *neutral*, *agree*, and *strongly agree*. The higher the score was, the higher the level of innovation behavior of the enterprise employees. The internal consistency coefficient of the scale was 0.844. The results of confirmatory factor analysis showed that all the fitting indexes were within the acceptable range, and the scale had good structural validity. According to the reliability and validity test, the Cronbach’s coefficient α was 0.854, and each test question was significantly correlated with the total score at the level of 0.01 (bilateral).

#### LMX Scale

The LMX scale (Odoardi et al., 2019) revised by Wang et al. (2014) according to the actual situation in China was adopted to evaluate the LMX. The scale contained four dimensions of emotion, loyalty, contribution, and professional respect, and provided a total of 16 test items, each of which had four items. Likert 5-grade rating system was adopted, and 1–5 points, respectively indicated *strongly disagree*, *disagree*, *neutral*, *agree*, and *strongly agree*. The higher the score was, the better the LMX was. The internal consistency coefficients of emotion, loyalty, contribution, and professional respect with the overall scale were 0.784, 0.816, 0.797, and 0.862, respectively. According to the reliability and validity test, the Cronbach’s coefficient α was 0.890, and each test question was significantly correlated with the total score at the level of 0.01 (bilateral).

### Statistical Analysis

SPSS19.0 statistical software was used for data processing in this study, and the measurement data was expressed as mean ± standard deviation (x ± s). The *t*-test of independent sample was used to analyze the differences of leaders’ psychological capital, employees’ innovative behavior, and exchange relationship between leaders and members in different genders and marriages. Also, group *t*-test was used to analyze the differences of leaders’ psychological capital, employees’ innovative behavior, and exchange relationship between leaders and members in different educational backgrounds and ages. In addition, Spearman correlation and multiple regression were used to analyze the pairwise relationship between leader’s psychological capital, employee innovation behavior, and leader member exchange relationship. Furthermore, hierarchical regression method was used to analyze the mediating effect of the dimensions of emotion, loyalty, and contribution on the psychological capital of leadership, emotion, and employee innovation.

## Results

### Analysis of Differences Among Demographic Variables

#### Analysis of Gender Differences

**FIGURE 3:**
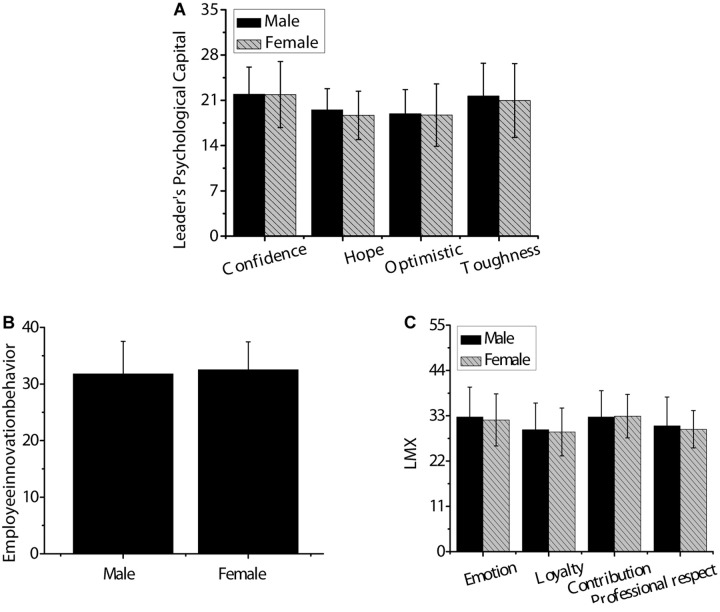
The difference of psychological capital, employee’s innovation behavior, and LMX among leaders of different genders. Panel **(A)** was confidence, hope, optimism, and resilience; panel **(B)** was innovative behavior of employees; panel **(C)** was affection, loyalty, contribution, and professional respect.

As shown in Figure 3, *t*-test was used to analyze the differences in psychological capital, employee innovation behavior, and LMX among leaders of different genders. There was no significant difference between men and women in each dimension of leaders’ psychological capital, employees’ innovation behavior, and LMX (*p* > 0.05).

#### The Difference Analysis of Different Marital Status

**FIGURE 4:**
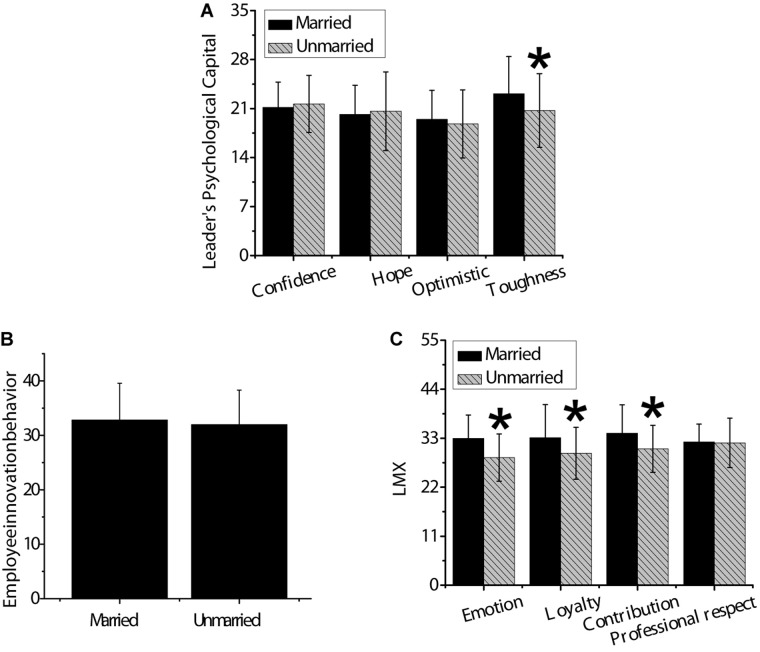
Differences in psychological capital of leaders, innovation behavior of employees, LMX with different marital status. Panel **(A)** was confidence, hope, optimism, and resilience; panel **(B)** was innovative behavior of employees; panel **(C)** was affection, loyalty, contribution, and professional respect. * indicated that compared with married people, the difference was statistically significant (*p* < 0.05).

As shown in Figure 4, *t*-test was used to analyze the differences in psychological capital of leaders with different marital status, innovation behavior of employees, and LMX. The score of married people in the dimension of leader’s psychological capital toughness was significantly higher than that of unmarried people (*p* < 0.05). The scores of emotion, loyalty, and contribution of married people were significantly higher than that of unmarried people (*p* < 0.05). There was no significant difference in confidence, optimism, hope, employee innovation behavior, and professional respect between married and unmarried people (*p* > 0.05).

#### Difference Analysis of Different Educational Background

**FIGURE 5:**
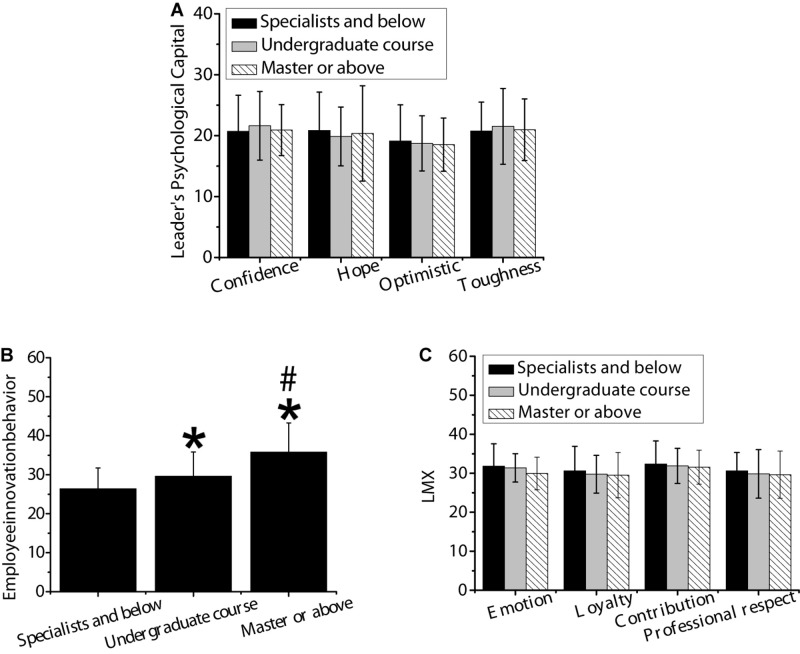
Differences in psychological capital, employee innovation behavior, and LMX of leaders with different educational degrees. Panel **(A)** was confidence, hope, optimism, and resilience; panel **(B)** was innovative behavior of employees; panel **(C)** was affection, loyalty, contribution, and professional respect. * indicated that the difference was statistically significant compared with master’s degree and below (*p* < 0.05). # indicated that there was a statistically significant difference compared with bachelor’s degree (*p* < 0.05).

As shown in Figure 5, variance test was used to analyze the differences in psychological capital, employee innovation behavior, and LMX with different educational degrees. There was no significant difference in the psychological capital of leaders and LMX with different educational degrees (*p* > 0.05). Moreover, the evaluation of innovation behavior of employees with a master’s degree or above was significantly higher than that with a bachelor’s degree or less, and the difference was statistically significant (*p* < 0.05). What’s more, the evaluation of innovation behavior of employees with a bachelor’s degree was significantly higher than that with a college degree or less, and the difference was statistically significant (*p* < 0.05).

#### Analysis of Age Differences

**FIGURE 6:**
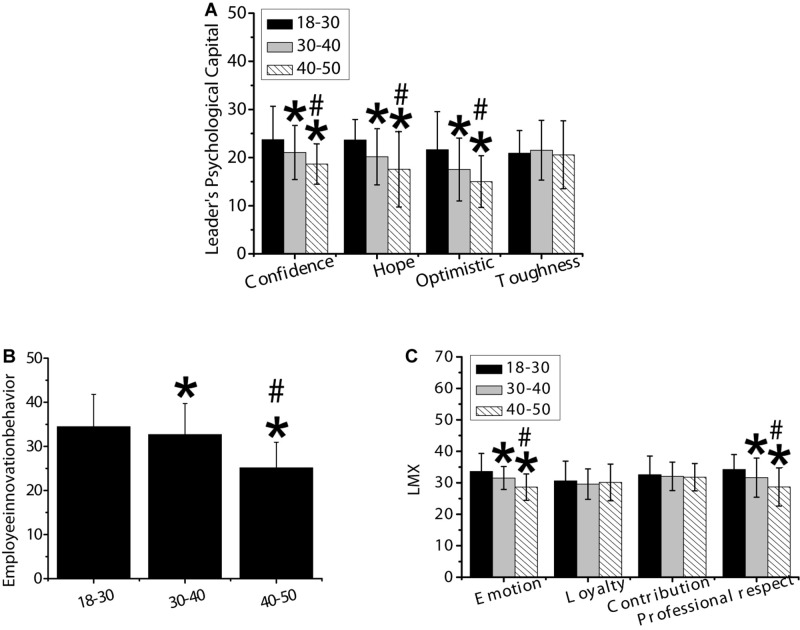
Differences in psychological capital, employee innovation behavior, and LMX of leaders with different ages. Panel **(A)** was confidence, hope, optimism, and resilience; panel **(B)** was innovative behavior of employees; panel **(C)** was affection, loyalty, contribution, and professional respect. * meant that the difference was statistically significant compared with those aged 18–30 (*p* < 0.05); # indicated that there was a statistically significant difference compared with those aged 30–40 (*p* < 0.05).

As shown in Figure 6, variance test was used to analyze the differences of psychological capital, employee innovation behavior, and LMX of different ages. The psychological capital of leaders aged 18–30 was significantly higher in the dimensions of confidence, hope, and optimism than those aged more than 30, and the difference was statistically significant (*p* < 0.05). The innovation behavior of employees aged 18–30 years was significantly higher than those aged more than 30 years, and the difference was statistically significant (*p* < 0.05). The LMX aged 18–30 was significantly higher than those aged more than 30 in terms of affection and professional respect, and the difference was statistically significant (*p* < 0.05).

### Correlation Analysis Between Variables

#### The Correlation Between Leader’s Psychological Capital and Employee’s Innovation Behavior

Table 3 shows the Spearman correlation analysis of four dimensions of confidence, hope, optimism, and resilience of leaders’ psychological capital and employee innovation behavior. There was a significant positive correlation between the total score of psychological capital, self-confidence, and toughness of leaders and the innovative behavior of employees (*p* < 0.001). In addition, the dimensions of hope and optimism were positively correlated with employee innovation behavior (*p* < 0.05).

**TABLE 3:** Spearman correlation between each dimension of leader’s psychological capital and employee’s innovation behavior.

Variables	Confidence	Hope	Optimism	Toughness	Total score of psychological capital
Innovation behavior of employees	0.769**	0.511*	0.471*	0.648**	0.539**

** *p* < 0.001, * *p* < 0.05.

#### The Correlation Between Psychological Capital of Leaders and LMX

Table 4 is the Spearman correlation analysis between each dimension of leaders’ psychological capital and LMX. The total score, confidence, optimism, and toughness of leaders’ psychological capital were positively correlated with emotion, loyalty, contribution, and professional respect of LMX (*p* < 0.001). The hope dimension was positively correlated with LMX dimensions of affection, loyalty, contribution, and professional respect (*p* < 0.05).

**TABLE 4:** Spearman correlation between leader’s psychological capital and LMX.

Variables	Confidence	Hope	Optimism	Toughness	The total score of psychological capital
Emotion	0.615**	0.538*	0.479**	0.632**	0.832**
Loyalty	0.534**	0.614*	0.531**	0.582**	0.518**
Contribution	0.672**	0.458*	0.628**	0.607**	0.677**
Professional respect	0.648**	0.516*	0.711**	0.514**	0.652**

** *p* < 0.001, * *p* < 0.05.

#### Correlation Between Employee Innovation Behavior and LMX

Table 5 is the Spearman correlation analysis of LMX and employee innovation behavior. There was a significant positive correlation between LMX emotion, loyalty, and contribution and employee innovation behavior (*p* < 0.001). The result shows that LMX professional respect was positively correlated with employee innovation behavior (*p* < 0.05).

**TABLE 5:** Spearman correlation between LMX and employee innovation behavior.

Variables	Emotion	Loyalty	Contribution	Professional respect
Innovation behavior of employees	0.639**	0.587*	0.782*	0.437*

** *p* < 0.001, * *p* < 0.05.

### Regression Analysis Between Variables

#### Regression Analysis of Leader’s Psychological Capital and Employee’s Innovation Behavior

As shown in Table 6, the multivariate regression analysis was conducted taking dimensions of psychological capital of leaders – confidence, hope, optimism, and toughness – as independent variables, and age (2 for 18–30 years old, 1 for 30–40 years old, and 0 for 40–50 years old), gender (1 for male, and 0 for female), marital status (1 for married, and 0 for unmarried), educational background (2 for master’s degree or above, 1 for bachelor’s degree, and 0 for junior college or below) as the control variables, and employee innovation behavior as an independent variable. Confidence and toughness had a very significant positive correlation with employee innovation behavior (*p* < 0.001). Hope and optimism were positively correlated with employee innovation behavior (*p* < 0.05). The control variables of age and educational background had significant predictive effects on employees’ innovation behavior (*p* < 0.05).

**TABLE 6:** Regression analysis of leader’s psychological capital and employee’s innovation behavior.

Variable		Regression coefficient	*T* value	*p*	*F* value	*R*^2^
Dependent variables	Confidence	0.731	5.518	0.000	163.750	0.816
	Hope	0.482	5.783	0.008		
	Optimism	0.407	3.638	0.014		
	Toughness	0.631	5.952	0.000		
Control variables	Gender	0.326	2.116	0.085		
	Age	0.471	5.376	0.015		
	Educational degree	0.539	4.842	0.023		
	Marriage	0.117	2.628	0.056		

#### The Regression Analysis of LMX and Employee Innovation Behavior

As shown in Table 7, LMX emotion, loyalty, contribution, and professional respect were taken as independent variables, the age, gender, marital status, and educational background as control variables, and employee innovation behavior as dependent variables for multivariate regression analysis. Contribution had a very significant positive correlation with employee innovation behavior (*p* < 0.001). Emotion and loyalty had significant positive correlation with employees’ innovation behavior (*p* < 0.05). The control variables age and educational background had significant predictive effects on employees’ innovation behavior (*p* < 0.05).

**TABLE 7:** The regression analysis of LMX and employee innovation behavior.

Variable		Regression coefficient	*T* value	*p*	*F* value	*R*^2^
Variable	Emotion	0.518	4.852	0.011	247.710	0.648
	Loyalty	0.573	5.892	0.008		
	Contribution	0.508	4.576	0.000		
	Professional respect	0.231	3.152	0.063		
Control variable	Gender	0.284	1.776	0.061		
	Age	0.452	5.376	0.008		
	Educational degree	0.631	5.717	0.016		
	Marriage	0.255	1.562	0.052		

#### The Regression Analysis of Leadership Psychological Capital and LMX

As shown in Table 8, the dimensions of psychological capital confidence, hope, optimism, and toughness of leaders were taken as independent variables, and age, gender, marital status, and educational background were taken as control variables. LMX total score was taken as dependent variables for multiple regression analysis. Confidence, hope, optimism, and resilience were significantly positively correlated with LMX (*p* < 0.001). Age and educational background were significant predictors of LMX (*p* < 0.05).

**TABLE 8:** The regression analysis of leadership psychological capital and LMX.

Variable		Regression coefficient	*T* value	*P*	*F* value	*R*^2^
Variable	Confidence	0.711	6.472	0.000	183.550	0.748
	Hope	0.684	6.735	0.000		
	Optimism	0.716	5.084	0.000		
	Toughness	0.752	5.873	0.000		
Control variable	Gender	0.310	2.416	0.052		
	Age	0.663	4.572	0.012		
	Educational degree	0.539	6.731	0.017		
	Marriage	0.164	1.742	0.067		

### The Mediating Effect of LMX in Leading Psychological Capital and Employee Innovation Behavior

According to the requirements of this study, the hierarchical regression method (Hussain and Shahzad, 2019) was used to test the mediating effect of LMX on the psychological capital of leaders and the innovation behavior of employees. The regression analysis revealed that the dimension of professional respect did not have a significant positive correlation with the innovation behavior of employees (*p* > 0.05), so only the dimensions of emotion, loyalty, and contribution needed to be analyzed as mediating variables.

#### Mediating Effect Analysis of Emotion

As shown in Table 9, with employee innovation behavior as the dependent variable, emotion and psychological capital of leaders were put into the regression equation for analysis. The psychological capital and emotion of leaders had a very significant positive influence on the innovation behavior of employees (*p* < 0.001), and the significance level of the model remained the same.

**TABLE 9:** Hierarchical regression analysis of mediating effect of emotion.

**Variable**		**Regression coefficient**	***T* value**	***p***	***F* value**	***R*^2^**
Variable	Psychological capital	0.538	6.165	0.000	159.660	0.185
Mediating variable	Emotion	0.517	5.742	0.000		
Control variable	Gender	0.261	2.852	0.057		
	Age	0.577	4.888	0.009		
	Educational degree	0.482	6.152	0.016		
	Marriage	0.162	1.592	0.074		

#### Mediating Effect Analysis of Loyalty

As shown in Table 10, with employees’ innovation behavior as the dependent variable, loyalty and psychological capital of leaders were put into the regression equation for analysis. The psychological capital and loyalty of leaders had a very significant positive effect on the innovation behavior of employees (*p* < 0.001), and the significance level of the model remained the same.

**TABLE 10:** Hierarchical regression analysis of the mediating effect of loyalty.

**Variable**		**Regression coefficient**	***T* value**	***p***	***F* value**	***R*^2^**
Independent variable	Psychological variable	0.672	5.382	0.000	241.770	0.152
Mediating variable	Loyalty	0.582	5.886	0.000		
Control variable	Gender	0.172	2.173	0.062		
	Age	0.573	6.582	0.012		
	Educational degree	0.516	6.537	0.011		
	Marriage	0.168	1.431	0.054		

#### Mediating Effect Analysis of Contributions

As shown in Table 11, with the innovation behavior of employees as the dependent variable, the contribution and the psychological capital of leaders were put into the regression equation for analysis. The psychological capital and contribution of leaders had a very significant positive effect on the innovation behavior of employees (*p* < 0.001), and the significance level of the model remained the same.

**TABLE 11:** Hierarchical regression analysis of mediating effects of contributions.

**Variable**		**Regression coefficient**	***T* value**	***p***	***F* value**	***R*^2^**
Independent variable	Psychological capital	0.822	7.472	0.000	247.720	0.167
Mediating variable	Contribution	0.662	6.728	0.000		
Control variable	Gender	0.199	1.528	0.085		
	Age	0.641	4.729	0.017		
	Educational degree	0.551	6.076	0.025		
	Marriage	0.175	1.468	0.056		

## Discussion

Nowadays, the competition among enterprises is extremely fierce. In order to stand firm in the industry and even take the lead, it is necessary to attach importance to innovation. Among them, the innovation of employees is one of the important driving forces for the development of enterprises, and it is also a research hotspot in organizational behavior. It is also the focus of many scholars to guide employees’ innovation behavior by relying on the improvement of leaders’ psychological capital (Mohamad et al., 2019). Therefore, the staff of several enterprises in a high-tech region were analyzed. Firstly, in terms of demographic variables, the scores of the psychological capital resilience and LMX affection, loyalty, and contribution of married leaders were significantly higher than those of unmarried leaders (*p <* 0.05). This was consistent with the research results of Jnaneswar (2019), indicating that married people were more able to bear the work due to the impact of family responsibilities and pay more attention to the existing job opportunities. However, there was no significant difference in the innovation behavior of employees (*p* > 0.05). The reason may be that the innovation behavior of employees was more dependent on personal professional knowledge, skills, and thinking ability, and can’t be changed simply because of marriage, which was consistent with the actual situation (Javed et al., 2019). The evaluation of innovation behavior of employees with a master’s degree or above was significantly higher than that with a bachelor’s degree or less (*p* < 0.05). This was completely contrary to the conclusion of Eisenberger et al. (2019) that employees with bachelor’s degrees had higher innovation ability than those with master’s degree. The reason for the analysis may be that the enterprises selected in this study were mainly in the Internet industry, and the employees with master’s degree or above undertook more sophisticated tasks and had higher innovation. Psychological capital (confidence, hope, optimism), employee innovation behavior, and LMX (emotion, professional respect) of leaders aged 18–30 were higher than those aged more than 30 (*p* (<0.05). This was because 18–30 year olds paid more attention to their relationship with their leader and had a better perception of innovation.

In order to analyze the relationship between LMX, psychological capital of leaders, and innovation behavior of employees, it was found using multiple regression analysis that confidence and resilience had a very significant positive correlation with innovation behavior of employees (*p* < 0.001). Hope and optimism had a significant positive correlation with employee innovation behavior (*p* < 0.05). This was consistent with the results of Özsungur (2019) indicating that the psychological capital of leaders had a significant positive influence on the innovation behavior of employees, and the influence of confidence and toughness was relatively big. Contribution had a very significant positive correlation with employee innovation behavior (*p* < 0.001). Emotion and loyalty had a significant positive correlation with employee innovation behavior (*p* < 0.05), which was the same as the research results of Opoku et al. (2020), indicating LMX was a significant positive predictor of employee innovation behavior, and its contribution was the strongest. On the other hand, there was no significant correlation between professional respect and employee innovation behavior (*p* > 0.05). The reason may be that professional respect was more reflected in the recognition and support of employees to leaders and professional competence of enterprises (Jung and Yoon, 2019). Confidence, hope, optimism, and resilience were significantly positively correlated with LMX (*p* < 0.001), indicating that leadership psychological capital had a significant predictive effect on LEX. In addition, the hierarchical regression method was also used in this study to test the mediating effect of LMX on the psychological capital of leaders and the innovation behavior of employees (Wang et al., 2019). The results showed that the dimensions of emotion, loyalty, and contribution had a major mediating effect between the psychological capital of leaders and the innovative behavior of employees, which indicated that situational factors related to leaders can have a potential impact on the effectiveness of leaders. The interaction between leaders and employees was actually the process of leadership. There were differences in the quality of leader-member relationships. High-quality relationships can have a positive impact, while low-quality relationships can have a negative impact, so relationship-oriented relationships may be a double-edged sword. Therefore, leaders should not only use psychological capital to promote employees’ innovative behaviors but also pay attention to the maintenance of good relationships among employees. To sum up, the relationship model of a leader’s psychological capital, LMX relationship, and employee’s innovation behavior established in this study was valid.

The fundamental purpose of academic research was to apply theories to real life. Based on the above analysis result, starting from the enterprise management level and actual demand, and aiming at coordinating the enterprise interior relationship, some suggestions were put forward to promote employee innovative behavior. At the organizational level, attention should be paid to the establishment of a harmonious, positive, and coordinated exchange relationship between leaders and members. The relationship between leaders and members will directly affect the daily operation of an enterprise. In daily work, it is advisable to organize more group building activities, strengthen the relationship between leaders and enterprise members outside work, expand the communication channels between employees and leaders, and at the same time pay attention to create an atmosphere conducive to innovative work. At the individual level, leaders should pay attention to the cultivation of their own interpersonal skills, and especially in the communication of employees, leaders should treat each employee with an equal attitude, actively abandon the concept of hierarchy, and try their best to support and understand each other with employees, so as to promote the smooth development of work. For employees, they also need to respect the daily work of the leader and treat the leader with a natural attitude. At the same time, they should actively seek for innovation consciousness to develop their brain, cultivate their own innovation consciousness in practice, get rid of the dependence on the supervision of the organization, so as to show more innovative behaviors.

## Conclusion

The hierarchical regression method was used to analyze the relationship between LMX, psychological capital of leaders, and innovation behavior of employees. It was found that demographic variables such as age and educational background has significant predictive effects on employee innovation behavior. The psychological capital of leaders not only has a significant positive effect on employee innovation behavior directly but also produces an indirect positive effect on employee innovation behavior by maintaining high-quality LMX. However, this study mainly adopts a horizontal research to explore the mediating role of LMX in the psychological capital of leadership and the innovation behavior of employees, and the influence of time effect is ignored. Therefore, longitudinal studies of a certain time span should be added to enrich the reliability of the conclusions in the future studies. In sum, this study provides a theoretical basis for leaders to manage enterprise talents and promote the practical operation of employees’ innovation ability.

## Data Availability Statement

The raw data supporting the conclusions of this article will be made available by the authors, without undue reservation.

## Ethics Statement

The studies involving human participants were reviewed and approved by the Shandong Agriculture and Engineering University and Wonkwang University Ethics Committees. The patients/participants provided their written informed consent to participate in this study.

## Author Contributions

All authors listed have made a substantial, direct and intellectual contribution to the work, and approved it for publication.

## Conflict of Interest

The authors declare that the research was conducted in the absence of any commercial or financial relationships that could be construed as a potential conflict of interest.
